# Prognostic tools for hypertrophic scar formation based on fundamental differences in systemic immunity

**DOI:** 10.1111/exd.14139

**Published:** 2020-08-17

**Authors:** Erik de Bakker, Mirthe A. M. van der Putten, Martijn W. Heymans, Sander W. Spiekstra, Taco Waaijman, Liselotte Butzelaar, Vera L. Negenborn, Vivian K. Beekman, Erman O. Akpinar, Thomas Rustemeyer, Frank B. Niessen, Susan Gibbs

**Affiliations:** ^1^ Department of Plastic, Reconstructive and Hand Surgery Amsterdam UMC, Vrije Universiteit Amsterdam Amsterdam Movement Sciences Amsterdam The Netherlands; ^2^ Department of Molecular Cell Biology and Immunolog Amsterdam UMC, Vrije Universiteit Amsterdam Amsterdam Movement Sciences Amsterdam The Netherlands; ^3^ Department of Plastic surgery OLVG Hospital Amsterdam The Netherlands; ^4^ Department of Epidemiology and Biostatistics Amsterdam UMC The Netherlands; ^5^ Department of Dermatology Amsterdam UMC, Vrije Universiteit Amsterdam Amsterdam Movement Sciences Amsterdam The Netherlands; ^6^ Department of Oral Cell Biology Academic Centre for Dentistry Amsterdam (ACTA), University of Amsterdam and Vrije Universiteit Amsterdam Amsterdam Movement Sciences Amsterdam The Netherlands

**Keywords:** cytokine, inflammation, prognostic, skin, wound healing

## Abstract

Unpredictable hypertrophic scarring (HS) occurs after approximately 35% of all surgical procedures and causes significant physical and psychological complaints. Parallel to the need to understanding the mechanisms underlying HS formation, a prognostic tool is needed. The objective was to determine whether (systemic) immunological differences exist between patients who develop HS and those who develop normotrophic scars (NS) and to assess whether those differences can be used to identify patients prone to developing HS. A prospective cohort study with NS and HS groups in which (a) cytokine release by peripheral blood mononuclear cells (PBMC) and (b) the irritation threshold (IT) after an irritant (sodium lauryl sulphate) patch test was evaluated. Univariate regression analysis of PBMC cytokine secretion showed that low MCP‐1, IL‐8, IL‐18 and IL‐23 levels have a strong correlation with HS (*P* < .010‐0.004; AUC = 0.790‐0.883). Notably, combinations of two or three cytokines (TNF‐a, MCP‐1 and IL‐23; AUC: 0.942, Nagelkerke R^2^: 0.727) showed an improved AUC indicating a better correlation with HS than single cytokine analysis. These combination models produce good prognostic results over a broad probability range (sensitivity: 93.8%, specificity 86.7%, accuracy 90,25% between probability 0.3 and 0.7). Furthermore, the HS group had a lower IT than the NS group and an accuracy of 68%. In conclusion, very fundamental immunological differences exist between individuals who develop HS and those who do not, whereas the cytokine assay forms the basis of a predictive prognostic test for HS formation, the less invasive, easily performed irritant skin patch test is more accessible for daily practice.

**Abbreviations**
AUC: area under the curve
HS: hypertrophic scar
IT: irritation threshold
LPS‐PG: lipopolysaccharide from Porphyromonas gingivalis
NS: normotrophic scar
PBMC: peripheral blood mononuclear cells
ROC: receiver operating characteristic

## 1 INTRODUCTION

Hypertrophic scarring is one of the most common complications of all surgeries. It is estimated that about 35% of surgical skin wounds heal with a hypertrophic scar (HS).^[^ ^1^ ^]^ Patients often experience a loss of quality of life due to physical or psychological complaints, especially when the scar is positioned over a joint.^[^ ^2^, ^3^ ^]^ Hypertrophic scarring is defined as a scar raised above the skin level because of excessive collagen deposition resulting in a scar that is thick, non‐pliable, itchy and painful.^[^ ^4^ ^]^ As opposed to a keloid, a hypertrophic scar remains within the confines of the wound. It is thought that the pathological mechanisms of these aberrant scars differ, and subsequently, the way they are managed also differs.^[^ ^3^, ^5^, ^6^, ^7^, ^8^ ^]^ Much research has been focussed on discovering the pathophysiology of these scars and treatment modalities. Parallel to the need to know the processes underlying HS formation, there is a need for a prognostic tool. This would have great value in the clinic, as an early start of treatment is preferred in HS.^[^ ^6^ ^]^ Being able to predict who is at risk to develop HS before going into surgery can help to prevent significant patient comorbidity and loss of quality of life as well as enabling better expectation management. In the case of elective, and in particular aesthetic surgery, patients and surgeons might choose to opt‐out of surgery when the benefit of the concerned procedure does not outweigh the chance of developing HS.

Many risk factors, like wound location, tension and mechanical loading, young age and bacterial colonization, have been identified for the development of HS.^[^ ^3^, ^8^, ^9^, ^10^ ^]^ However, it is still unknown why one individual will develop an HS after surgery whereas another will not.^[^ ^7^, ^9^ ^]^ The immune system is increasingly seen as essential in answering the questions surrounding HS formation.^[^ ^11^ ^]^ HS formation is associated with increased numbers of inflammatory cells like mast cells and epidermal Langerhans cells and increased levels of cytokines like IL‐4.^[^ ^7^, ^12^, ^13^ ^]^ Lower levels of cytokines within the wound and scar area, such as IL‐1α, IL‐6, IL‐8 and CXCL‐8, suggest a reduced inflammatory response may be responsible.^[^ ^14^, ^15^ ^]^ Considering the pivotal role of blood‐derived mononuclear cells (eg monocytes and lymphocytes) in regulating the early inflammatory phase once these cells infiltrate the wound bed,^[^ ^13^, ^16^ ^]^ a systemic origin of the early immunological differences between normal scars (NS) and HS should be considered. Such a difference would provide both prognostic markers and targets for the development of therapeutic agents.

Notably, many of the cytokines which are regulated in the skin during HS formation are also released during skin irritation, which can be seen as microtrauma.^[^ ^15^, ^17^ ^]^ Skin irritation, and thus the induction of an innate immune response, can easily be tested by determining the irritation threshold (IT) of a topically applied substance. It has been described that a range of ITs exists within a group of people with regard to their responsiveness to topically applied sodium lauryl sulphate (SLS) in a patch test.^[^ ^18^ ^]^ Therefore, assuming a different (early) immune response between NS and HS formers, it would seem logical to hypothesize that the IT could be a discriminating factor between these two groups as well, and it could then subsequently be used as a predictive tool. Determining the skin IT by means of a patch test with an irritant is a non‐invasive, easily applicable test with low intra‐individual variation.^[^ ^19^, ^20^ ^]^

Considering this background, a prospective cohort study was performed with both an NS and HS group in which both the IT after an irritant SLS patch test and cytokine release by peripheral blood mononuclear cells (PBMC) were evaluated. Furthermore, transepidermal water loss (TEWL) and cytokines present within stratum corneum tape strips obtained from the patch test sites were assessed. Cytokine bead‐based immunoassays for analysing samples were chosen after considering which cytokines would be expected to be secreted from PBMCs and which may be expected to be detectable from stratum corneum tape strips. As an example, this was based on our previous research where we showed that MCP‐1 is only secreted at very low levels by epidermal keratinocytes but was secreted in high levels by monocytes.^[^ ^21^ ^]^ This study aimed to determine whether (systemic) immunological differences exist between patients who develop HS and those who develop normotrophic scars (NS) and to assess whether those differences can be used to identify patients prone to developing HS.

## 2 MATERIALS AND METHODS

### 2.1 Patient inclusion

Between 2014 and 2016, 31 patients were included in this prospective observational cohort study (see flow chart depicting the inclusion, Figure [Link], [Link]). This study was performed in accordance with the Declaration of Helsinki and the guidelines for Good Clinical Practice. The independent medical ethics review boards of the participating hospitals approved the study protocol (https://www.toetsingonline.nl, number NL40722.029.13). Written informed consent was obtained from all participants. All patients volunteered to participate in this study and were healthy, adult females who had undergone reduction mammoplasty more than 5 months (average 11 months) prior to inclusion. The mammoplasty scars were evaluated in this study. Patients were excluded if their skin type, skin condition, medical treatment or unwillingness to adhere to life rules during the study would impede the results of patch testing (Table 1). After physical examination by an experienced plastic surgeon (FBN), the volunteers were divided into an NS group and an HS group, assigned a patient number for subsequent anonymized (blinded; not performed by FBN) data processing of IT, peripheral blood and TEWL, and baseline characteristics were collected (Table 2). Scars were scored normotrophic if they were flat at the level of the surrounding tissue and coloured like the surrounding tissue. Scars were considered hypertrophic when red and raised at least 2 mm above the skin level. All mammoplasty procedures were performed in a standard fashion. The surgical wounds were closed in layers with the cutaneous closure using absorbable intra‐cutaneous suture material.

**TABLE 1:** Exclusion criteria and life rules

Fitzpatrick photo skin types V and VI
Skin disease, for example psoriasis, pemphigus vulgaris etc
Skin lesions, tattoos or substantial hair growth patch test site
NS group: thickening of scars at any time after surgery
Pregnancy/lactation during the first 2 years postoperatively or the patch test
Topical immunosuppressive treatment of the upper arm in the last 7 days before the patch test
Application of skin lotions/ointments on the upper arm in the last 6 weeks before the patch test
Considerable exposure of the upper arm to UVR in the last 14 days before the patch test
Systemic antibiotic treatment in the last 2 wk before patch test
Systemic immunosuppressive treatment during the first 2 years after surgery or in the last 6 months before the patch test
Immunological disorders: infectious disease, immune deficiencies, auto‐immune disorders
Alcohol or drug abuse
Smoking during the first two postoperative years
ASA classification 3 or higher
Participation in another clinical study
Performing physical activities which cause heavy sweating, sauna, swimming or extreme showers or baths during the study

Note

Exclusion criteria and life rules.

**TABLE 2:** Patient characteristics

	NS (n = 15)	HS (n = 16)
Age (years)	50 (10)	48 (8)
BMI (kg/m^2^)	25.5 (3.3)	27.7 (4.0)
HS at other site	1/15	2/16
Atopic	67%	75%
Past smoker	42%	40%
Fitzpatrick Skin type		
I	0	1
II	6	5
III	6	6
IV	3	4
V	0	0
VI	0	0

Note

Patient characteristics at inclusion. Mean or percentage ± SD (standard deviation) is shown.

### 2.2 Cytokine secretion profile of peripheral blood mononuclear cells

Peripheral blood mononuclear cells (PBMC) were isolated from blood collected at inclusion using Lymphoprep^tm^ (Stemcell Technologies) according to manufacturers' instructions. Cells were frozen in liquid nitrogen until use. PBMC were thawed and cultured in Iscove's Modified Dulbecco's Medium (IMDM) with 1% penicillin/streptomycin; 1% glutamine (100 mmol/L) and 5 μmol/L B‐mercaptoethanol at 37°C at 5% CO_2_. In a 96‐well plate, 2 × 10^5^ PBMC were seeded per well and stimulated with 0, 1, 3.3 or 10 μg/mL lipopolysaccharide (LPS) from Porphyromonas gingivalis (LPS‐PG, InvivoGen) for 48 hours. LPS was chosen in this study as it is frequently used as a positive control when testing substances which may stimulate an immune response in PBMCs, since being of bacterial origin it stimulates the innate and adaptive immune systems.^[^ ^21^, ^22^ ^]^ Plates were centrifuged at 300 g for 5 minutes, and the supernatant was harvested and stored at −20°C. The supernatant was analysed using a bead‐based immunoassay from BioLegend LEGENDplex (BioLegend). The human inflammation panel (IL‐1β, IFN‐α2, IFN‐γ, TNF‐α, MCP‐1, IL‐6, IL‐8, IL‐10, IL‐12p70, IL‐17A, IL‐18, IL‐23 and IL‐33) was used according to the manufacturer's instructions. Samples were diluted by a factor of 50.

### 2.3 SLS irritation patch testing

Patch testing was performed on the non‐dominant upper arm with the application of the contact irritant sodium lauryl sulphate (SLS) (0%, 0.25%, 0.5%, 1% and 2% in water). SLS is used routinely in skin patch tests in both research and clinical practice since it penetrates the stratum corneum, as opposed to LPS which is a large bacterial membrane molecule. Van der Bend^®^ patch test chambers on Fixomull^®^ tape were filled with 20 μL of test solution. The patch was removed 48 hours later by the participants themselves. On day four, the test was assessed by an experienced dermatologist (TR). The lowest concentration of SLS which induces an irritation reaction is the IT. The amount of irritation is graded using the visual grading scale for irritation (Table 3).^[^ ^23^ ^]^ The percentage of SLS and the corresponding patch test gradings between the NS and HS group were then processed using ROC analysis to determine which percentage of SLS could best discriminate between the two groups in the test.

**TABLE 3:** Visual grading scale for irritation

Score	Irritation reaction
0	No visible reaction
1	Tobacco paper‐like appearance, no erythema
2	Slight patchy erythema
3	Homogeneous erythema
4	Erythema with oedema
5	Erythema, oedema and vesicles/bulla

Note

Adopted from Basketter et al^[^ ^23^ ^]^.

Furthermore, non‐invasive measurement of transepidermal water loss (TEWL) by means of a TEWAmeter^®^ (TM300; Courage & Khazaka) was performed on the patch test sites to assess skin barrier disruption, which is a parameter for skin irritation.^[^ ^16^ ^]^ TEWL was performed following established guidelines, with the patient resting for 10 minutes in a room free of excessive draughts, and stable temperature and moisture.^[^ ^16^ ^]^ Two readings (in g/m^2^h) were taken from normal skin on the arm and each of the patch test sites.^[^ ^24^ ^]^ Measurement of skin redness by means of a DermaSpectrometer^®^ (Cortex Technology) was performed.^[^ ^25^ ^]^ The probe was placed on the skin and the erythema index (E = 100×log[intensity of reflected red light/intensity of reflected green light]) was determined as well as a melanin index (M = 100×log[1/intensity of reflected red light]). The E parameter is used for the evaluation of vascularization and the M parameter for pigmentation. Stratum corneum was collected via tape‐stripping of the patch test sites and was analysed for cytokine secretion as explained in Figure [Link], [Link].

### 2.4 Statistical analysis

Sodium lauryl sulphate patch test: it was expected that 70% of the HS group would have a high IT compared to 30% of the NS group. For clinical relevance, this means that patients with a high IT will have a 70% risk of hypertrophic scar formation. In practice, a high IT means that a person will respond with the same skin reaction to a higher dose of SLS compared to someone with a normal IT. A sample size of 30 patients per group was calculated. Thirty patients in each group would be enough to reject the null hypothesis that the probability of skin irritation for the two groups is equal with a probability (power) of 0.8. The type I error probability associated with this test of this null hypothesis is 0.05. After including 30 patients, an interim analysis was performed, and due to the ample significance in this analysis, the study was concluded. Two‐way ANOVA followed by Sidak's multiple comparison test and ROC analysis were performed using GraphPad Prism version 7.00 for Windows, GraphPad Software, www.graphpad.com. A *P*‐value of <.05 was considered statistically significant. This method of analysis gives a yes‐no answer as to whether an individual is prone to developing a hypertrophic scar.

Peripheral blood mononuclear cells cytokine test: Two‐way ANOVA was used for all cytokines, a *P*‐value of <.05 was considered statistically significant (GraphPad Prism). To form a prediction model on the PBMC data, binary logistic regression analysis was performed and a ROC curve calculated (IBM SPSS Statistics for Windows, version 22.0). Single cytokine and cytokine combinations were analysed in the same fashion. The data generated by measuring cytokine secretion facilitate this type of analysis. The advantage is that all data are retained in the analysis as opposed to setting a certain threshold. The disadvantage of this technique is that it means that the PBMC model and patch test model cannot be directly compared with each other. After individual analysis for each cytokine, those with a large area under the curve (AUC) and significant *P*‐value were selected to create combinations with optimum prognostic value. These combinations were filtered in a similar manner. The probability of a hypertrophic scar forming can then be calculated with the formula: p = 1/(1+*ϵ* ^−^ *^LP^*) where*LP* = constant + (coefficient × cytokine1) + (coefficient × cytokine2). The cytokine input is either pg/mL or ng/mL. Internal validation procedures were used to represent how the model would perform in new patients and to compensate for overfitting of the model on the data (RStudio Team 2015) RStudio, Inc).

## 3 RESULTS

Patient characteristics are shown in Table 2. NS was observed in 15 patients and HS in 16 patients. The baseline characteristics show two groups without large discrepancies. One outlier is a patient who developed a normotrophic scar at her mammoplasty site with a formerly hypertrophic scar elsewhere on her body (her knee) which was a flat scar at the moment of inclusion.

### 3.1 Differential cytokine secretion in hypertrophic scar compared to normotrophic scar patients

**FIGURE 1:**
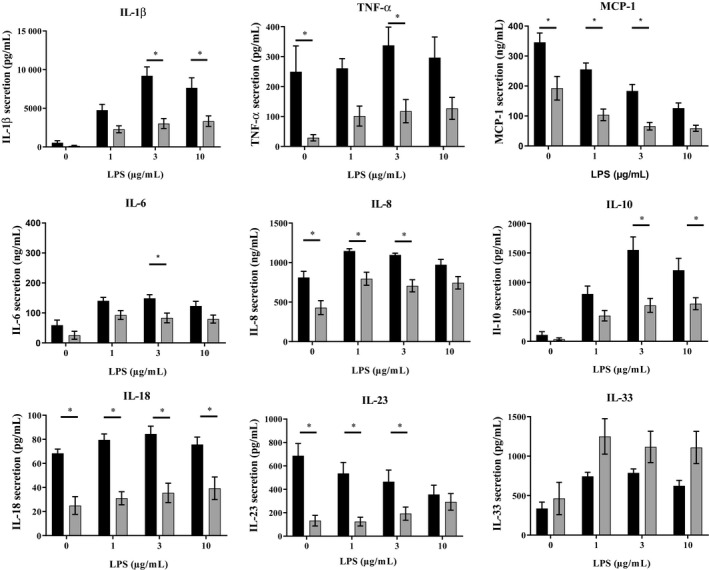
Cytokine secretion. Cytokine secretion by peripheral blood mononuclear cells (PBMCs) after being cultured for 48 h and stimulated with increasing concentrations of LPS. Normotrophic patients (n = 15) = bars black, hypertrophic patients (n = 16) = grey bars. Mean ± SEM (standard error of the mean) is shown. A *P*‐value of <.05 was considered statistically significant

The secretion of TNF‐α, MCP‐1, IL‐8, IL‐18 and IL‐23 by unstimulated PBMC was significantly lower in the HS group compared to the NS group (Figure 1). No significant difference in the secretion of IL‐1β, IL‐6, IL‐10 and IL‐33 by unstimulated PBMCs was observed, while secretion of IFN‐α2, IFN‐γ, IL‐12p70 and IL‐17A remained below the detection threshold of the assay.

In order to mimic an inflammatory response, PBMC were stimulated with LPS. An increase in secretion was observed for IL‐1β, TNF‐a, IL‐6, IL‐8, IL‐10 and IL‐33 in both NS and HS patients compared to unstimulated PBMC. The secretion of IL‐18 was unaltered in both groups, whereas MCP‐1 secretion decreased in both groups. IL‐23 secretion decreased in a dose‐dependent manner in the NS group after stimulation while secretion increased in the HS group until secretion from the NS and HS groups was at the same level at LPS 10 μg/mL stimulation (Figure 1). Notably, for IL‐6, IL‐8 and IL‐23, the fold increase compared to unstimulated PBMC was greater in the HS group compared to the NS group even though total protein remained lower in the HS group compared to the NS group (Figure 1 and Figure [Link], [Link]), for example 1 µg/mL LPS results in IL‐6:7.2‐fold increase in NS group compared to 66.2‐fold increase in HS group, IL‐8:1.7‐fold increase in NS group compared to 3.3‐fold increase in HS group and IL‐23:0.8‐fold decrease in NS group compared to 56.5‐fold increase in HS group at 2 µg/mL LPS.

### 3.2 Prediction model based on unstimulated PBMC cytokine secretion

The cytokine secretion by unstimulated PBMC was further used to develop a prediction model. The results of the univariate regression analysis of unstimulated PBMC cytokine secretion are shown in Table 4. MCP‐1, IL‐8, IL‐18 and IL‐23 have a strongly significant correlation with HS (*P* < .010‐0.004). TNF‐α, which was significant in 2‐way ANOVA, now showed a strong trend (*P* < .060). All of the five cytokines showed a good area under the curve (AUC = 0.790‐0.883), confidence interval, odds ratio and Nagelkerke R^2^ (see Table 5). Next, based on the merit of the AUC and *P‐*values of the individual cytokines, all possible combinations of these five cytokines were analysed (Tables 4, 5, 6, Table S1). Notably, combinations of 2 cytokines (MCP‐1 and IL‐23; AUC: 0.921, Nagelkerke R^2^: 0.703) or 3 cytokines (TNF‐a, MCP‐1 and IL‐23; AUC: 0.942, Nagelkerke R^2^: 0.727) showed clearly improved AUC indicating a better correlation with HS than single cytokine analysis (Tables 4, 5, 6). As expected with these AUC values, these combination models produce excellent prognostic results (eg sensitivity, specificity and accuracy) over a broad probability range (sensitivity: 93.8%, specificity 86.7%, accuracy 90.25% between probability 0.3 and 0.7) (Table 6; Table S1 for all cytokines combinations). Combinations of more than three cytokines had no further added value.

**TABLE 4:** Univariate regression analysis of unstimulated PMBC cytokine secretion

Type		Coefficient	OR	95% CI	Nagelkerke	*P*	AUC	95% CI
IL‐1β	Pg/mL	−0.002	0.998	0.994‐1.001	0.164	.174	0.825	0.670‐0.980
**TNF‐α**	**Pg/mL**	−**0.014**	**0.986**	**0.972‐1.001**	**0.357**	**.060**	**0.820**	**0.667‐0.973**
**MCP‐1**	**Ng/mL**	−**0.008**	**0.992**	**0.986‐0.998**	**0.338**	**.010**	**0.796**	**0.633‐0.959**
IL‐6	Ng/mL	−0.001	0.999	0.992‐1.007	0.001	.873	0.750	0.562‐0.938
**IL‐8**	**Ng/mL**	−**0.003**	**0.997**	**0.994‐0.999**	**0.354**	**.007**	**0.812**	**0.663‐0.962**
IL‐10	Pg/mL	−0.004	0.996	0.989‐1.003	0.077	.285	0.667	0.467‐0.866
**IL‐18**	**Pg/mL**	−**0.073**	**0.930**	**0.885‐0.977**	**0.597**	**.004**	**0.883**	**0.746‐1.000**
**IL‐23**	**Pg/mL**	−**0.006**	**0.994**	**0.990‐0.998**	**0.562**	**.007**	**0.867**	**0.736‐0.997**
IL‐33	Pg/mL	0.000	1.000	0.999‐1.002	0.017	.553	0.404	0.190‐0.619

Note

Binary logistic regression analysis of unstimulated PBMC cytokine secretion after 48 hr culture identifies patients with a hypertrophic scar. MCP‐1, IL‐8, IL‐18, IL‐23 and TNF‐α were selected to form stronger combinations on the merit of strong AUC and *P‐*value. Cytokines in bold text were used for combination analysis

Abbreviations: AUC, area under the curve; CI, confidence interval; coefficient for the constant; Nagelkerke R^2^; OR, odds ratio; prob, probability.

**TABLE 5:** Prognostic performance of cytokine combinations

Cytokines	Constant	Coefficient	OR	95% CI	Nagelkerke	*P*	AUC	95% CI	Ad. AUC	Ad. Nagelkerke
Group N = 2	4.58				0.703		0.921	0.811‐1.0	0.91	0.66
MCP‐1		−0.009	0.991	0.982‐1.000		0.041				
IL‐23		−0.005	0.995	0.991‐0.999		0.008				
Group N = 3	4.713				0.727		0.942	0.857‐1.0	0.92	0.63
TNF‐α		−0.006	0.994	0.976‐1.012		0.496				
MCP‐1		−0.009	0.991	0.982‐1.000		0.048				
IL‐23		−0.004	0.996	0.992‐0.999		0.022				
Group N = 4	4.704				0.728		0.942	0.855‐1.0	0.90	0.56
IL‐8		0.001	1.001	0.994‐1.008		0.802				
IL‐23		−0.004	0.996	0.992‐0.999		0.022				
MCP‐1		−0.011	0.989	0.973‐1.006		0.194				
TNF‐α		−0.007	0.993	0.974‐1.012		0.459				
Group N = 5	4.813				0.731		0.947	0.867‐1.0	0.87	0.46
IL‐8		0.001	1.001	0.993‐1.008		0.856				
IL‐18		−0.011	0.989	0.931‐1.051		0.721				
IL‐23		−0.004	0.996	0.992‐1.000		0.08				
MCP‐1		−0.009	0.991	0.973‐1.008		0.299				
TNF‐α		−0.007	0.993	0.973‐1.014		0.522				

Note

Prognostic performance of cytokine combinations. Internal validation procedures were used to represent how the model would perform in new patients and to compensate for overfitting of the model on the data (RStudio Team [2015]. RStudio, Inc,). See Materials and Methods, section Statistical Analysis.

Abbreviations: AUC, area under the curve; CI, confidence interval; coefficient for the constant; Nagelkerke R^2^; OR, odds ratio; prob, probability.

**TABLE 6:** Prognostic characteristics of cytokine prediction models

MCP‐1 + IL23							
Prob	Sens	Spec	Acc	FN	FP	PPV	NPV
0.1	93.8	46.7	70.25	1	8	65.2	87.5
0.2	93.8	80	86.9	1	3	83.3	92.3
0.3	93.8	86.7	90.25	1	2	88.2	92.9
0.4	93.8	86.7	90.25	1	2	88.2	92.9
0.5	93.8	86.7	90.25	1	2	88.2	92.9
0.6	93.8	86.7	90.25	1	2	88.2	92.9
0.7	87.5	93.3	90.4	2	1	93.3	87.5
0.8	68.8	93.3	81.05	5	1	91.7	73.7
0.9	43.8	100	71.9	9	0	100	62.5

TNF‐α + MCP‐1 + IL23							
Prob	Sens	Spec	Acc	FN	FP	PPV	NPV
0.1	93.8	46.7	70.25	1	8	65.2	87.5
0.2	93.8	66.7	80.25	1	5	75	90.9
0.3	93.8	86.7	90.25	1	2	88.2	92.9
0.4	93.8	86.7	90.25	1	2	88.2	92.9
0.5	93.8	86.7	90.25	1	2	88.2	92.9
0.6	93.8	86.7	90.25	1	2	88.2	92.9
0.7	93.8	93.3	93.55	1	1	93.8	93.3
0.8	62.5	93.3	77.9	6	1	90.3	70
0.9	43.8	100	71.9	9	0	100	62.5

Note

Prognostic characteristics of cytokine prediction models at increasing probability of developing a hypertrophic scar.

Abbreviations: acc, accuracy; coefficient for the constant; FN, false negative; FP, false positive; NPV, negative predictive value; PPV, positive predictive value; prob, probability; sens, sensitivity; spec, specificity.

### 3.3 Prediction model based on irritation threshold

**FIGURE 2:**
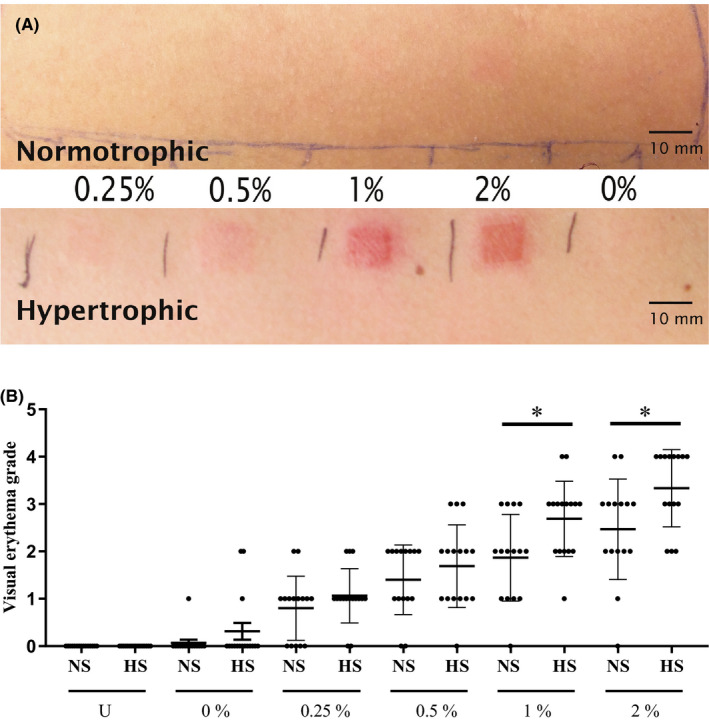
Patch test results. A, Representative results of reaction to SLS patch testing in an HS and a NS patient. SLS concentrations of 0.25%, 0.5%, 1%, 2% and 0% in water. The duration of the patch test was 48 h and the readout was at 96 h. Erythema grades given were 0 (0.25%, 1 (0.5%), 2 (1%), 2 (2%) and 0 (0%) for the NS patient and 2 (0.25%, 3 (0.5%), 4 (1%), 4 (2%) and 0 (0%) for the HS patient, see grading scale in Table 3 B, Scatter plot with standard error of the mean (SEM) of all visual erythema grading scores of unexposed skin (U) and patch test sites exposed to percentage of SLS in water. Black bar = HS, grey bar = NS. Normotrophic patients (n = 15) = bars black, hypertrophic patients (n = 16) = grey bars. 2‐way ANOVA performed in GraphPad Prism

In both patient groups, increasing visual erythema correlated to increasing SLS concentration with the dose‐dependent increase in patch test score being greater for HS than for NS (Figure 2A). Notably, the difference between NS and HS was significant for SLS 1% (*P* < .027; range patch test score: NS 0‐3 vs HS 1‐4) and 2% (*P* < .014; range patch test score: NS 0‐4 vs HS 2‐4) concentrations indicating that the HS group has a lower IT than the NS group (Figure 2B). In Figure 2B, all visual erythema grading scale scores are visualized with a scatter plot. After ROC curve analysis following 2‐way ANOVA, both 1% and 2% SLS showed good discriminatory characteristics when using a cut‐off of ≥2 on the visual irritation scale. With a 1% SLS solution, sensitivity and specificity are 60%and 73%, respectively, with an overall accuracy of 66.7%. Increasing the percentage to 2% results in higher sensitivity (80%) while lowering the specificity (46.7%), with an overall accuracy of 63.3%. The grading scores at concentrations of SLS lower than 1% were too similar between the two groups to be useful (see Figure 2B). No significant difference were observed between the control (vehicle water) exposed sites of the NS and HS groups which both scored negative (0) according to the visual irritation grading scale (Table 3).

The normotrophic patient with a formerly hypertrophic scar on her knee interestingly had a non‐conclusive score in both patch testing (scoring visual erythema grades of 2 for 1% and 3 for 2% SLS) as well as in the cytokine panel (lower than average for NS secretion of TNF‐α [48.5 vs 249.5 pg/mL], MCP‐1 [120.0 vs 345.8 ng/mL], IL‐6 [6.0 vs 59.1 ng/mL] and IL‐8 [242.0 vs 811.5 ng/mL]).

In contrast to the visual irritation grading, neither the TEWL nor the dermatospectrometry measurements were able to distinguish the HS from the NS group based on IT (Figure [Link], [Link]). Cytokines extracted from the stratum corneum also did not show significant differences between the two groups (see Figure [Link], [Link]).

## 4 DISCUSSION

Here, we show that very fundamental immunological differences exist between individuals who develop HS and those who do not. More importantly, the suppressed PBMC cytokine secretion observed in HS individuals compared to NS individuals is the basis of a novel predictive prognostic test for HT formation. The less invasive and easily performed irritant skin patch test showed a differentiating induction response in HS individuals compared to NS individuals and therefore provides a more accessible option for daily practice.

Of the 13 cytokines studied in the stimulated PBMC assay, 5 cytokines (TNF‐α, MCP‐1, IL‐8, IL‐18 and IL‐23) had potential prognostic value due to the clearly lower levels detected in unstimulated PBMC cultures derived from HS individuals compared to NS individuals. These individual cytokines had, in logistic regression analysis, an AUC ranging from 0.796 to 0.883. When combining these individual cytokines into a panel of two (MCP‐1 and IL‐23) or three (add TNF‐α) cytokines, the AUC increased to 0.921 and 0.942, respectively, while the accuracy of these combinations was >90% over a large range. All of these are excellent values for a prognostic tool.

Many studies implicate the roles of TNF‐α, MCP‐1, IL‐6 and IL‐8 in wound healing. MCP‐1, IL‐6 and IL‐8 have been described to be a chemoattractant for monocytes and neutrophils which regulate the inflammatory phase of wound closure.^[^ ^26^, ^27^ ^]^ All four are mitogens and stimulate re‐epithelialization.^[^ ^28^ ^]^ In contrast, IL‐18 and IL‐23 are not well‐known cytokines in wound regulation. IL‐18 has been associated with (cutaneous) inflammatory skin diseases like psoriasis, allergic contact dermatitis and atopic dermatitis,^[^ ^29^, ^30^ ^]^ in addition to keloid formation.^[^ ^31^ ^]^ It is produced by keratinocytes and plays a key role in innate immunity and inflammasome activation, although its role in wound healing has not yet been reported.^[^ ^32^ ^]^ IL‐23 is closely related to IL‐18 and can be produced by activated macrophages and dendritic cells^[^ ^33^ ^]^; it has been linked to autoimmunity and is involved in the differentiation of Th17 cells.^[^ ^32^, ^34^, ^35^ ^]^ In line with our current findings, we previously described a local suppressed inflammatory mRNA expression (TNF‐α, IL‐1α, IL‐1RN, CCL2, CCL3, CXCL2, CXCR2, C3 and IL‐10) within the early healing wound and the young scar over a 52‐week follow‐up period as well as significantly lower concentrations of inflammatory proteins in the postsurgical wound site in hypertrophic scars.^[^ ^15^, ^36^ ^]^ This suggested a reduced inflammatory response which conflicted with the existing belief that HS is related to increased inflammation.^[^ ^15^, ^37^, ^38^ ^]^ Despite the low basal cytokine secretion in HS individuals, when PBMCs were stimulated, the fold increase in IL‐6, IL‐8 and IL‐23 cytokine secretion was greater than in NS individuals. IL‐23 is of particular interest as the HS group shows a 56.2‐fold increase, whereas the NS group actually shows a small 0.8‐fold decrease. Importantly, this increase in secretion did not result in a higher absolute secretion compared to NS patients and would therefore still present as a reduced inflammatory response when comparing NS and HS directly. HS patients have in a resting state a lowered inflammatory cytokine profile, but upon triggering, a clear cytokine response is initiated. Further studies are required to determine whether the low basal cytokine secretion followed by a potential increased inflammatory reaction is also a contributory factor to HS formation.

Our finding that a functional difference of the immune system at a systemic level is an important factor in HS formation is a critically different approach to focussing on the biology within the local site of injury. While acknowledging risk factors like age and allergy status,^[^ ^3^, ^8^, ^9^, ^10^ ^]^ there is a group of individuals who are susceptible to developing HS despite these risk factors and who will be predisposed to develop HS regardless. The immune suppression which we describe in this study may now be considered as a potent risk factor. This further expands the puzzle of HS formation in which we know that injury in the deep dermis is predictive for HS development as well as the fact that scars are often partially hypertrophic and normotrophic.^[^ ^39^ ^]^ Our results showed that both unstimulated and stimulated PBMCs from the HS group secreted lower amounts of IL‐10 than the NS group. IL‐10 is a potent anti‐inflammation regulatory cytokine and has been described extensively in the context of hypertrophic scar formation and therefore has generated interest as a potential anti‐scarring agent.^[^ ^36^, ^40^ ^]^ Although further research is required, these first results may provide a link between HS formation and the more inflammatory irritation patch test result that was observed in the HS group.

Although some of the results described in our study and the existing body of research seem contradictory, most of the current literature focuses on the local site of injury as opposed to these systemic processes.^[^ ^41^, ^42^, ^43^, ^44^ ^]^ Indeed, a different population of immune cells are present in the skin (eg Langerhans cells, macrophages and resident T cells) compared to their counterparts in the blood since immune cell plasticity and the influence of the micro‐environment determine immune cell phenotype and their cytokine secretome.^[^ ^45^ ^]^ This may be the reason that we observe an increased inflammatory response in HS patients upon a localized skin irritant challenge and a decreased baseline cytokine secretion from PBMCs in the same HS group. This finding cannot be considered a discrepancy since two totally different stand‐alone methods were used, one which assesses a local skin immune reaction and the other a systemic peripheral blood immune reaction. To our knowledge, there are no publications describing an irritant patch test response for other forms of fibrosis, for example in keloid patients or the stimulation of keloid‐derived PBMCs with LPS. However, it has been described that IL‐18 secretion was reduced in both unstimulated and LPS‐stimulated monocytes from patients with atopic dermatitis.^[^ ^46^ ^]^ This is an interesting subject for a follow‐up study as it will indeed determine whether our observations are specific for HS patients or more general for fibrosis and other inflammatory skin diseases, for example Rosacea, keratosis pilaris, eczema and psoriasis. Although, in the past, we have investigated IL‐6 and IL‐8 secretion from healthy and keloid‐derived monocytes and did not observe a decreased baseline secretion in the keloid monocytes.^[^ ^47^ ^]^ Also, TNF‐alpha and IL‐6 have also been reported to be increased in LPS‐stimulated healthy PBMC,^[^ ^48^ ^]^ although we only observed a moderate increase, and in contrast to our results, it has been described that LPS increases secretion of MCP‐1 from PBMCs derived from healthy donors.^[^ ^22^ ^]^ The expanding knowledge on the mechanisms of hypertrophic scar formation would seem to indicate that it is an intricate process in which individual cells and cytokines act in both stimulatory and suppressive roles at certain moments over the entire course of scar maturation, and therefore, this may also explain some of the discrepancies which we find with reports of others.^[^ ^15^, ^36^ ^]^ In our study, we only included mature NS and HS scars resulting from the same surgical intervention.

Although slightly less impressive, patch testing the skin with an irritant also resulted in clear differences between the NS and HS groups. It should be noted that this is a more subjective test, requiring an experienced dermatologist to assess the patch test sites. Based on earlier research describing cytokine mRNA expression in skin tissue biopsies, we had expected a higher IT, which would correlate with a suppressed local immune reaction to trauma in the HS group.^[^ ^15^, ^36^ ^]^ The fact that we observed a lower IT in HS individuals does, however, reflect the changes in the PBMC cytokine profiles where HS patients responded more substantially with a greater fold induction compared to NS individuals. Our patch test findings cannot be explained by HS individuals having an inferior barrier function compared to NS individuals since cytokines isolated from stratum corneum tape strips, visual erythema and TEWL showed no significant differences, indicating that the difference originates deeper in the skin.

In this study, we describe two prognostic tests to determine whether an individual may be prone to developing HS after surgery. The PBMC test reaches 93% accuracy; however, it is an invasive test that requires peripheral blood and expertise in cell culture and therefore is relatively time‐consuming to implement into routine procedures. The patch test, while being slightly less accurate (63%‐68%) than the PBMC test, has the advantage that it is minimally invasive and does not require a cell culture laboratory. However, it does require an experienced dermatologist to score the patch test. Further studies might well improve the accuracy and clinical usability of the patch test, for example by testing distinct more skin irritants, different concentrations and different time intervals.^[^ ^49^, ^50^ ^]^ Although both tests have their specific pros and cons, they do both provide easily accessible tools to estimate the chance of an individual developing HS after surgery. In addition to providing an option not to undergo surgery if it is not essential, extra attention can be given to patients at risk during and directly after surgery, for example by performing extra meticulous surgery and using reduced tension skin stitching methods, as well as the start of silicone application at two weeks following surgery. The PBMC cytokine secretion profiles need further confirmation in larger cohorts, but our results clearly suggest that these mediators can be critical predictors. Furthermore, these cytokines offer new insights into pathophysiological mechanisms and, hence, these findings stimulate future research into prevention and treatment.

These prognostic markers have proven strong in our cohort, but prospective research is needed in a larger group of patients including males and all skin types, including patients who have not yet developed a significant scar to further strengthen these results. Similar research should be considered in the keloid patient group considering the greater influence on the quality of life. Furthermore, if measuring cytokines directly in serum proves equally effective, the more time laborious process of PBMC isolation and culture could be omitted.

In conclusion, our results indicate that HS patients exhibit a systemically suppressed immune status with lower PBMC cytokine secretion. After stimulation, a more pronounced response than in NS patients is seen but still falls short of cytokine secretion of individuals who form NS. Ultimately, this may result in a failure to successfully complete the normal wound healing process. These findings enable a very potent model to predict the formation of hypertrophic scars.

## CONFLICT OF INTEREST

The authors declare no conflict of interest.

## AUTHOR CONTRIBUTION

FN, SG, MP, EB and LB conceptualized and designed the study. EB, SG, FN, MH, SS, TW and TR analysed the data. EB, MP and SG wrote the manuscript. MP and EB contributed to study coordination. EB, MP, VN, VB and EA collected data. All authors have read and approved the final manuscript.

## Supporting information


**Fig S1** Flow‐chart depicting the inclusion of patients

**Fig S2** Patch test TEWL and dermatospectrometry results

**Fig S3** Stimulation indexes for cytokine secretion

**Fig S4** Cytokine detection from patch test sites

**Table S1** prognostic characteristics of prediction models
