# Supplementary material for: Prognostic tools for hypertrophic scar formation based on fundamental differences in systemic immunity
Source: Exp Dermatol. 2020 Aug 17;30(1):169–78. doi: 10.1111/exd.14139 (PMC7818462; doi:10.1111/exd.14139)
Supplement: Supplementary file 1 — Fig S1 Flow‐chart depicting the inclusion of patients [file EXD-30-169-s001.pdf]

de Bakker et al. Supplementary figure 1

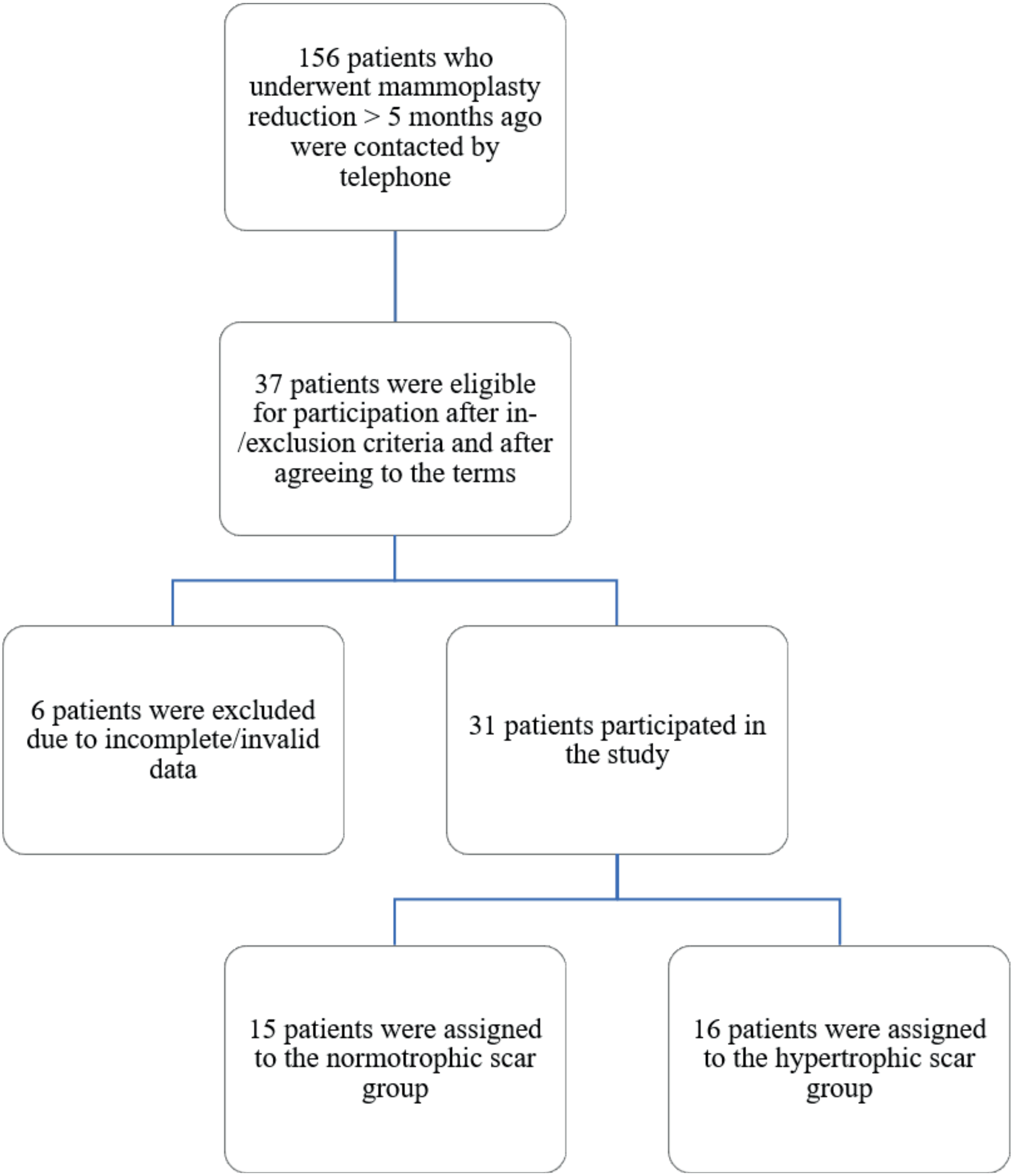

Supplement figure 1: flow-chart depicting the inclusion of patients

Exclusions: 1 patient did not adhere to the life rules, the others were excluded due to unsuccessful venipuncture at the start of the inclusion and/or an incorrectly prepared batch of SLS patch tests.
