# Supplementary material for: Prognostic tools for hypertrophic scar formation based on fundamental differences in systemic immunity
Source: Exp Dermatol. 2020 Aug 17;30(1):169–78. doi: 10.1111/exd.14139 (PMC7818462; doi:10.1111/exd.14139)
Supplement: Supplementary file 2 — Fig S2 Patch test TEWL and dermatospectrometry results [file EXD-30-169-s002.pdf]

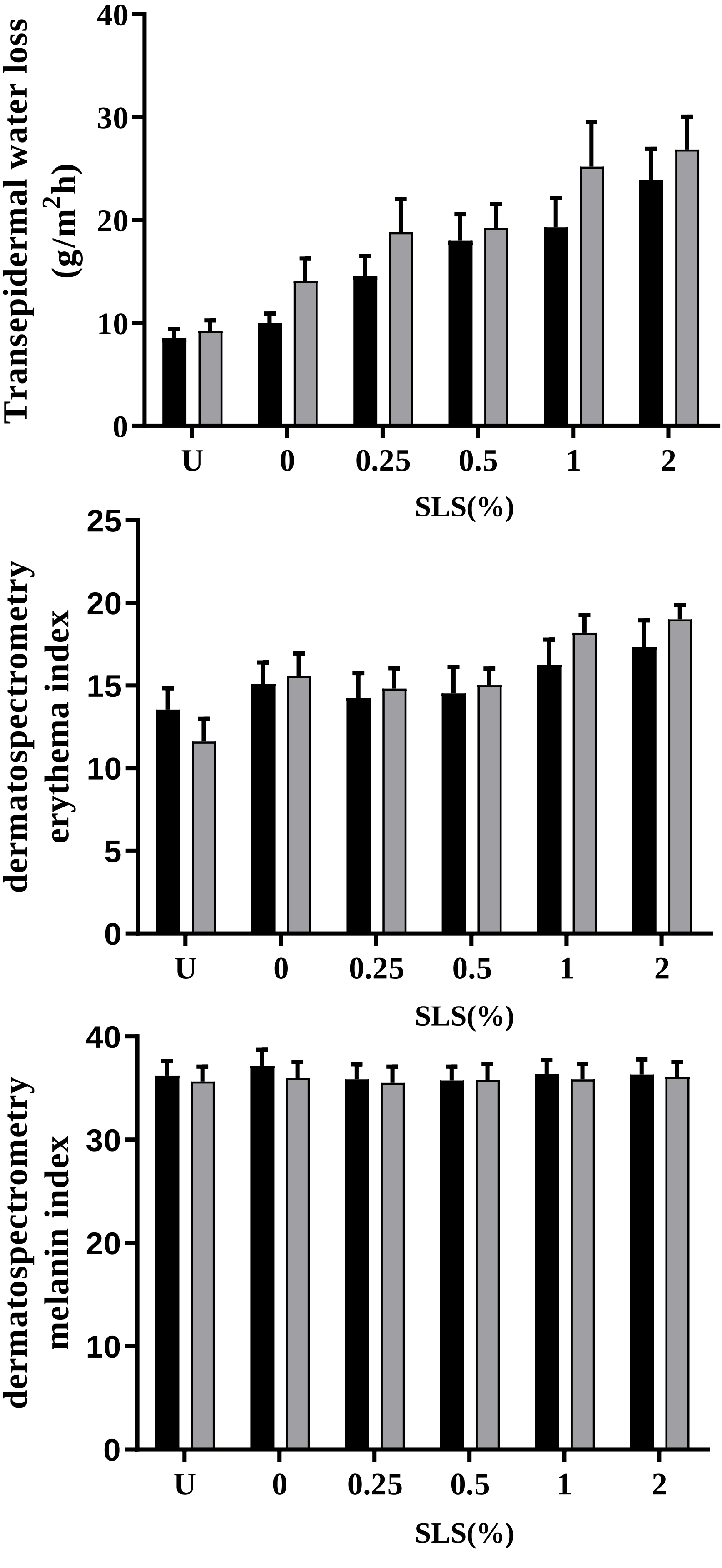

Supplement figure 2: patch test TEWL and dermatospectrometry results

Patch test results for unexposed skin (U) and patch test sites exposed to a percentage of SLS in aqua. Blackbar= normotrophic scar,gray bar = hypertrophic scar. a). Transepidermal water loss (TEWL) in g/m2 h. b) dermatospectrometry results. TEWL was measured using a TEWAmeter® (TM300; Courage &Khazaka, Cologne, Germany) on the patch test sites to assess skin barrier disruption, which is a parameter for skin irritation[16]. This was done following established guidelines, with the patient rested for 10 minutes in a room free of excessive draughts and a stable temperature16. Two readings (in g/m2h) were taken from normal skin of the opposite arm and each of the patch test sites15. Also, a measurement of skin redness (erythema index) utilizing a DermaSpectrometer® (Cortex Technology, Hadsund, Denmark) was performed[16]. The probe is placed on the skin and the device produces an erythema index (E=100× log(intensity of reflected red light/intensity of reflected green light) as well as a melanin index (M=100× log(1/intensity of reflected red light). The E parameter is used for the evaluation of vascularisation and the M parameter for pigmentation
