# Supplementary material for: Prognostic tools for hypertrophic scar formation based on fundamental differences in systemic immunity
Source: Exp Dermatol. 2020 Aug 17;30(1):169–78. doi: 10.1111/exd.14139 (PMC7818462; doi:10.1111/exd.14139)
Supplement: Supplementary file 3 — Fig S3 Stimulation indexes for cytokine secretion [file EXD-30-169-s003.pdf]

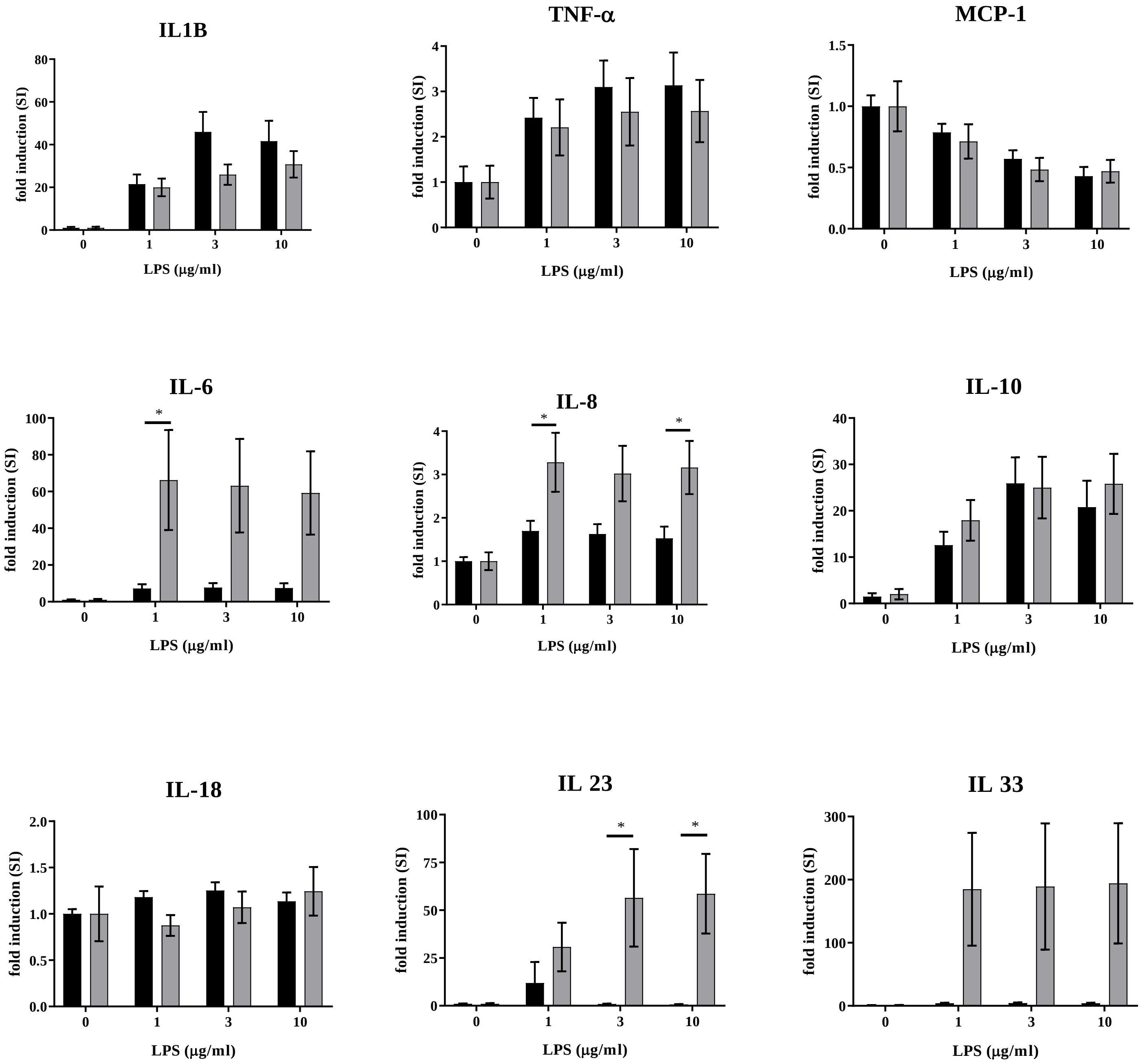

Supplement figure 3; stimulation indexes for cytokine secretion

Stimulation indexes of secretion of cytokines by peripheral mononuclear cells (PBMCs) after culture for 48 hours. Secretion after stimulation with increasing concentrations of LPS is shown relative to the unstimulated condition. Normotrophic patients (n=15) = black bars, hypertrophic patients (n=16) = gray bars. Mean +/- SEM (standard error of the mean) is shown. A p-value of <0,05 was considered statistically significant.
