# Supplementary material for: Prognostic tools for hypertrophic scar formation based on fundamental differences in systemic immunity
Source: Exp Dermatol. 2020 Aug 17;30(1):169–78. doi: 10.1111/exd.14139 (PMC7818462; doi:10.1111/exd.14139)
Supplement: Supplementary file 4 — Fig S4 Cytokine detection from patch test sites [file EXD-30-169-s004.pdf]

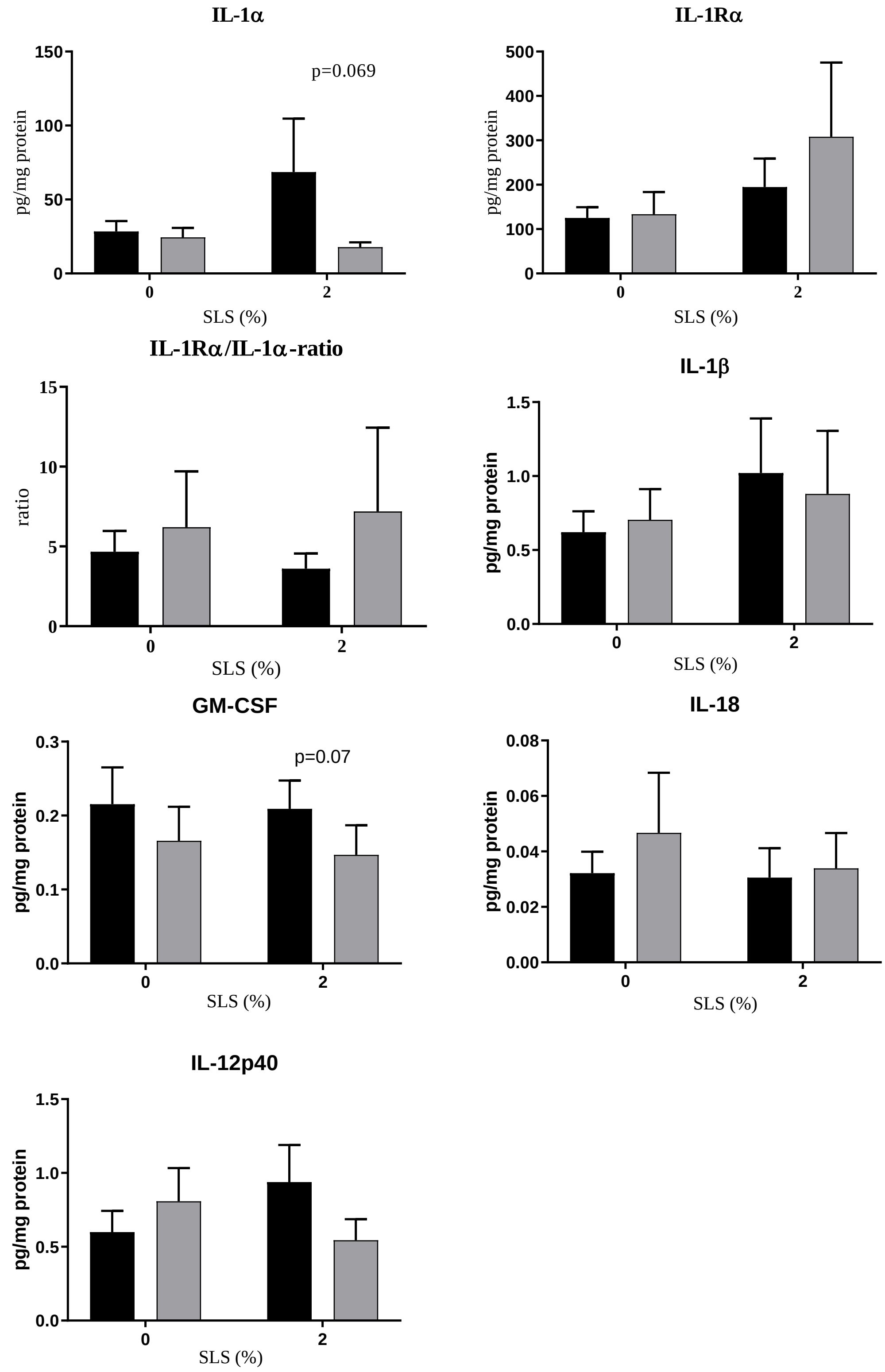

Supplement figure 4; cytokine detection from patch test sites.

Cytokine secretion measured in tape-stripped stratum corneum (SC) after 48 hours of exposure to 0% and 2% SLS in aqua.Normotrophic patients (n=15) = bars black, hypertrophic patients (n=16) = gray bars. SC was collected using Scotch Crystal tape for cytokine analysis as previously described (PMID: 17908190). In short, each patch site and one unexposed site was stripped five times after applying a roller for standardized pressure with two 19mm x20mm tapes. The tapes were pooled per site and after dip-freezing in liquid nitrogen stored at -80° Celsius. SC was then separated from the tapes by an ultrasound sonifier in 1 ml pbs +0.05% tween. Soluble protein was determined in the strips supernatant (Biorad protein assay, Hercules, Ca, USA). Both IL-1α and IL1-Rα were analysed by using specific enzyme-linked-immunosorbent assay (ELISA) kits (R&D Systems, Oxon, UK) Other cytokines were determined using the human cytokine panel 2 bead-based immunoassay from Biolegendlegendplex (BioLegend, San Diego, Ca., USA) used according to the manufacturer's instructions.This panel was chosen because it includes cytokines known to be found in the stratum corneum, such as IL-1α and GM-CSF. IFN-α2, IL-11, IL-15, IL-18, IL-23, IL-27, IL-33 and TSLP were below the detection limit in most samples. Two-way ANOVA analysis was performed using GraphPad Prism version 7.00 for Windows, GraphPad Software, La Jolla California USA, www.graphpad.com. No sample showed significant results.
