# Supplementary material for: Prognostic tools for hypertrophic scar formation based on fundamental differences in systemic immunity
Source: Exp Dermatol. 2020 Aug 17;30(1):169–78. doi: 10.1111/exd.14139 (PMC7818462; doi:10.1111/exd.14139)
Supplement: Supplementary file 5 — Table S1 prognostic characteristics of prediction models [file EXD-30-169-s005.docx]

**Appendix S1**

**Supplement Table 1**: prognostic characteristics of prediction models

| **MCP-1+IL23** | | | | | | | |  | **TNF-α +MCP-1+IL-18** | | | | | | | |
| --- | --- | --- | --- | --- | --- | --- | --- | --- | --- | --- | --- | --- | --- | --- | --- | --- |
| Prob | Sens | Spec | Acc | FN | FP | PPV | NPV |  | Prob | Sens | Spec | Acc | FN | FP | PPV | NPV |
| 0.1 | 93.8 | 46.7 | 70.25 | 1 | 8 | 65.2 | 87.5 |  | 0.1 | 100 | 33.3 | 66.65 | 0 | 10 | 61.5 | 100 |
| 0.2 | 93.8 | 80 | 86.9 | 1 | 3 | 83.3 | 92.3 |  | 0.2 | 87.5 | 53.3 | 70.4 | 2 | 1 | 66.7 | 80 |
| 0.3 | 93.8 | 86.7 | 90.25 | 1 | 2 | 88.2 | 92.9 |  | 0.3 | 87.5 | 60 | 73.75 | 2 | 6 | 70 | 81.8 |
| 0.4 | 93.8 | 86.7 | 90.25 | 1 | 2 | 88.2 | 92.9 |  | 0.4 | 87.5 | 80 | 83.75 | 2 | 3 | 82.4 | 85.7 |
| 0.5 | 93.8 | 86.7 | 90.25 | 1 | 2 | 88.2 | 92.9 |  | 0.5 | 87.5 | 93.3 | 90.4 | 2 | 1 | 93.3 | 87.5 |
| 0.6 | 93.8 | 86.7 | 90.25 | 1 | 2 | 88.2 | 92.9 |  | 0.6 | 75 | 100 | 87.5 | 4 | 0 | 100 | 78.9 |
| 0.7 | 87.5 | 93.3 | 90.4 | 2 | 1 | 93.3 | 87.5 |  | 0.7 | 75 | 100 | 87.5 | 4 | 0 | 100 | 78.9 |
| 0.8 | 68.8 | 93.3 | 81.05 | 5 | 1 | 91.7 | 73.7 |  | 0.8 | 75 | 100 | 87.5 | 4 | 0 | 100 | 78.9 |
| 0.9 | 43.8 | 100 | 71.9 | 9 | 0 | 100 | 62.5 |  | 0.9 | 62.5 | 100 | 81.25 | 6 | 0 | 100 | 71.4 |
|  | | | | | | | |  |  |  |  |  |  |  |  |  |
| **TNF-α +MCP-1+IL23** | | | | | | | |  | **TNF-α +IL8+IL23** | | | | | | | |
| Prob | Sens | Spec | Acc | FN | FP | PPV | NPV |  | Prob | Sens | Spec | Acc | FN | FP | PPV | NPV |
| 0.1 | 93.8 | 46.7 | 70.25 | 1 | 8 | 65.2 | 87.5 |  | 0.1 | 100 | 46.7 | 73.35 | 0 | 8 | 66.7 | 100 |
| 0.2 | 93.8 | 66.7 | 80.25 | 1 | 5 | 75 | 90.9 |  | 0.2 | 93.8 | 60 | 76.9 | 1 | 6 | 71.4 | 90 |
| 0.3 | 93.8 | 86.7 | 90.25 | 1 | 2 | 88.2 | 92.9 |  | 0.3 | 87.5 | 73.3 | 80.4 | 2 | 4 | 77.8 | 84.6 |
| 0.4 | 93.8 | 86.7 | 90.25 | 1 | 2 | 88.2 | 92.9 |  | 0.4 | 87.5 | 86.7 | 87.1 | 2 | 2 | 87.5 | 86.7 |
| 0.5 | 93.8 | 86.7 | 90.25 | 1 | 2 | 88.2 | 92.9 |  | 0.5 | 87.5 | 86.7 | 87.1 | 2 | 2 | 87.5 | 86.7 |
| 0.6 | 93.8 | 86.7 | 90.25 | 1 | 2 | 88.2 | 92.9 |  | 0.6 | 87.5 | 86.7 | 87.1 | 2 | 2 | 87.5 | 86.7 |
| 0.7 | 93.8 | 93.3 | 93.55 | 1 | 1 | 93.8 | 93.3 |  | 0.7 | 81.3 | 86.7 | 84 | 3 | 2 | 86.7 | 81.3 |
| 0.8 | 62.5 | 93.3 | 77.9 | 6 | 1 | 90.3 | 70 |  | 0.8 | 68.8 | 93.3 | 81.05 | 5 | 1 | 91.7 | 73.7 |
| 0.9 | 43.8 | 100 | 71.9 | 9 | 0 | 100 | 62.5 |  | 0.9 | 43.8 | 93.3 | 68.55 | 9 | 1 | 87.5 | 60.9 |
|  |  |  |  |  |  |  |  |  |  |  |  |  |  |  |  |  |
| **MCP-1+IL8+IL23** | | | | | | | |  | **MCP-1+IL18+IL23** | | | | | | | |
| Prob | Sens | Spec | Acc | FN | FP | PPV | NPV |  | Prob | Sens | Spec | Acc | FN | FP | PPV | NPV |
| 0.1 | 93.8 | 46.7 | 70.25 | 1 | 8 | 65.2 | 87.5 |  | 0.1 | 93.8 | 46.7 | 70.25 | 1 | 8 | 65.2 | 87.5 |
| 0.2 | 93.8 | 80 | 86.9 | 1 | 3 | 83.3 | 92.3 |  | 0.2 | 93.8 | 66.7 | 80.25 | 1 | 5 | 75 | 90.9 |
| 0.3 | 93.8 | 86.7 | 90.25 | 1 | 2 | 88.2 | 86.7 |  | 0.3 | 93.8 | 86.7 | 90.25 | 1 | 2 | 88.2 | 92.9 |
| 0.4 | 93.8 | 86.7 | 90.25 | 1 | 2 | 88.2 | 86.7 |  | 0.4 | 93.8 | 86.7 | 90.25 | 1 | 2 | 88.2 | 92.9 |
| 0.5 | 93.8 | 86.7 | 90.25 | 1 | 2 | 88.2 | 86.7 |  | 0.5 | 93.8 | 86.7 | 90.25 | 1 | 2 | 88.2 | 92.9 |
| 0.6 | 93.8 | 86.7 | 90.25 | 1 | 2 | 88.2 | 86.7 |  | 0.6 | 87.5 | 86.7 | 87.1 | 2 | 2 | 87.5 | 86.7 |
| 0.7 | 93.8 | 86.7 | 90.25 | 1 | 2 | 88.2 | 86.7 |  | 0.7 | 75 | 93.3 | 84.15 | 4 | 1 | 92.3 | 77.8 |
| 0.8 | 62.5 | 93.3 | 77.9 | 6 | 1 | 90.9 | 70 |  | 0.8 | 68.8 | 93.3 | 81.05 | 5 | 1 | 91.7 | 73.7 |
| 0.9 | 43.8 | 100 | 71.9 | 9 | 0 | 100 | 62.5 |  | 0.9 | 43.8 | 100 | 71.9 | 9 | 0 | 100 | 62.5 |
|  |  |  |  |  |  |  |  |  |  |  |  |  |  |  |  |  |
| **IL8+IL18+IL23** | | | | | | | |  | **IL8+IL18+ IL23+TNF-α** | | | | | | | |
| Prob | Sens | Spec | Acc | FN | FP | PPV | NPV |  | Prob | Sens | Spec | Acc | FN | FP | PPV | NPV |
| 0.1 | 100 | 53.3 | 76.65 | 0 | 7 | 69.6 | 100 |  | 0.1 | 100 | 53.3 | 76.65 | 0 | 7 | 69.6 | 100 |
| 0.2 | 93.8 | 66.7 | 80.25 | 1 | 5 | 75 | 90.9 |  | 0.2 | 93.8 | 66.7 | 80.25 | 1 | 5 | 75 | 90.9 |
| 0.3 | 93.8 | 73.3 | 83.55 | 1 | 4 | 78.9 | 91.7 |  | 0.3 | 93.8 | 73.3 | 83.55 | 1 | 4 | 78.9 | 91.7 |
| 0.4 | 93.8 | 86.7 | 90.25 | 1 | 2 | 88.2 | 92.9 |  | 0.4 | 93.8 | 86.7 | 90.25 | 1 | 2 | 88.2 | 92.9 |
| 0.5 | 87.5 | 86.7 | 87.1 | 2 | 2 | 87.5 | 86.7 |  | 0.5 | 87.5 | 86.7 | 87.1 | 2 | 2 | 87.5 | 86.7 |
| 0.6 | 75 | 86.7 | 80.85 | 4 | 2 | 85.7 | 76.5 |  | 0.6 | 75 | 86.7 | 80.85 | 4 | 2 | 85.7 | 76.5 |
| 0.7 | 68.8 | 93.3 | 81.05 | 5 | 1 | 91.7 | 73.7 |  | 0.7 | 75 | 93.3 | 84.15 | 4 | 1 | 92.3 | 77.8 |
| 0.8 | 62.5 | 93.3 | 77.9 | 6 | 1 | 90.9 | 70 |  | 0.8 | 68.8 | 93.3 | 81.05 | 5 | 1 | 91.7 | 73.7 |
| 0.9 | 56.3 | 100 | 78.15 | 7 | 0 | 100 | 68.2 |  | 0.9 | 62.5 | 100 | 81.25 | 6 | 0 | 100 | 71.4 |

| **IL8+IL18+IL23+MCP-1** | | | | | | | |  | **IL18+IL23+MCP-1+TNF-α** | | | | | | | |
| --- | --- | --- | --- | --- | --- | --- | --- | --- | --- | --- | --- | --- | --- | --- | --- | --- |
| Prob | Sens | Spec | Acc | FN | FP | PPV | NPV |  | Prob | Sens | Spec | Acc | FN | FP | PPV | NPV |
| 0.1 | 93.8 | 53.3 | 73.55 | 1 | 7 | 68.2 | 88.9 |  | 0.1 | 93.8 | 46.7 | 70.25 | 1 | 8 | 65.2 | 87.5 |
| 0.2 | 93.8 | 66.7 | 80.25 | 1 | 5 | 75 | 90.9 |  | 0.2 | 93.8 | 66.7 | 80.25 | 1 | 5 | 75 | 90.9 |
| 0.3 | 93.8 | 86.7 | 90.25 | 1 | 2 | 88.2 | 92.9 |  | 0.3 | 93.8 | 86.7 | 90.25 | 1 | 2 | 88.2 | 92.9 |
| 0.4 | 93.8 | 86.7 | 90.25 | 1 | 2 | 88.2 | 92.9 |  | 0.4 | 93.8 | 86.7 | 90.25 | 1 | 2 | 88.2 | 92.9 |
| 0.5 | 93.8 | 86.7 | 90.25 | 1 | 2 | 88.2 | 92.9 |  | 0.5 | 93.8 | 86.7 | 90.25 | 1 | 2 | 88.2 | 92.9 |
| 0.6 | 87.5 | 86.7 | 87.1 | 2 | 2 | 87.5 | 86.7 |  | 0.6 | 87.5 | 86.7 | 87.1 | 2 | 2 | 87.5 | 86.7 |
| 0.7 | 75 | 93.3 | 84.15 | 4 | 1 | 92.3 | 77.8 |  | 0.7 | 81.3 | 93.3 | 87.3 | 3 | 1 | 92.9 | 82.4 |
| 0.8 | 68.8 | 93.3 | 81.05 | 5 | 1 | 91.7 | 73.7 |  | 0.8 | 68.8 | 93.3 | 81.05 | 5 | 1 | 91.7 | 73.7 |
| 0.9 | 50 | 100 | 75 | 8 | 0 | 100 | 65.2 |  | 0.9 | 62.5 | 100 | 81.25 | 6 | 0 | 100 | 71.4 |
|  |  |  |  |  |  |  |  |  |  |  |  |  |  |  |  |  |
| **IL-8+IL23+MCP-1+TNF-α** | | | | | | | |  | **IL-8+IL18+IL23+MCP-1+TNF-α** | | | | | | | |
| Prob | Sens | Spec | Acc | FN | FP | PPV | NPV |  | Prob | Sens | Spec | Acc | FN | FP | PPV | NPV |
| 0.1 | 93.8 | 53.3 | 73.55 | 1 | 7 | 68.2 | 88.9 |  | 0.1 | 93.8 | 46.7 | 70.25 | 1 | 8 | 65.2 | 87.5 |
| 0.2 | 93.8 | 66.7 | 80.25 | 1 | 5 | 75 | 90.9 |  | 0.2 | 93.8 | 66.7 | 80.25 | 1 | 5 | 75 | 90.9 |
| 0.3 | 93.8 | 86.7 | 90.25 | 1 | 2 | 88.2 | 92.9 |  | 0.3 | 93.8 | 86.7 | 90.25 | 1 | 2 | 88.2 | 92.9 |
| 0.4 | 93.8 | 86.7 | 90.25 | 1 | 2 | 88.2 | 92.9 |  | 0.4 | 93.8 | 86.7 | 90.25 | 1 | 2 | 88.2 | 92.9 |
| 0.5 | 93.8 | 86.7 | 90.25 | 1 | 2 | 88.2 | 92.9 |  | 0.5 | 93.8 | 86.7 | 90.25 | 1 | 2 | 88.2 | 92.9 |
| 0.6 | 93.8 | 86.7 | 90.25 | 1 | 2 | 88.2 | 92.9 |  | 0.6 | 87.5 | 86.7 | 87.1 | 2 | 2 | 87.5 | 86.7 |
| 0.7 | 87.5 | 93.3 | 90.4 | 2 | 1 | 93.3 | 87.5 |  | 0.7 | 87.5 | 93.3 | 90.4 | 2 | 1 | 93.3 | 87.5 |
| 0.8 | 68.8 | 93.3 | 81.05 | 5 | 1 | 91.7 | 73.7 |  | 0.8 | 75 | 93.3 | 84.15 | 4 | 1 | 92.3 | 77.8 |
| 0.9 | 43.8 | 100 | 71.9 | 9 | 0 | 100 | 62.5 |  | 0.9 | 43.8 | 100 | 71.9 | 9 | 0 | 100 | 62.5 |

Prognostic characteristics (sensitivity, specificity, accuracy, false negative (FN), false positive (FP), negative predictive value (NPV), positive predictive value (PPV)) of cytokine combinations to predict hypertrophic scar presence at different probability cut-off points (prob.).
